# Autocrine DUSP28 signaling mediates pancreatic cancer malignancy via regulation of PDGF-A

**DOI:** 10.1038/s41598-017-13023-w

**Published:** 2017-10-06

**Authors:** Jungwhoi Lee, Jungsul Lee, Jeong Hun Yun, Chulhee Choi, Sayeon Cho, Seung Jun Kim, Jae Hoon Kim

**Affiliations:** 1 Department of biotechnology, College of Applied Life Science, SARI, Jeju National University, Jeju-do, 63243 Republic of Korea; 2 Department of Bio and Brain Engineering, KAIST, Daejeon, 34141 Republic of Korea; 3 College of Pharmacy, Chung-Ang University, Seoul, 06974 Republic of Korea; 4 Division of Strategic Research Planning and Assessment, Korea Research Institute of Bioscience & Biotechnology, Daejeon, 34141 Republic of Korea

## Abstract

Pancreatic cancer remains one of the most deadly cancers with a grave prognosis. Despite continuous efforts to improve remedial values, limited progress has been made. We have reported that dual specificity phosphatase 28 (DUSP28) has a critical role of chemo-resistance and migration in pancreatic cancers. However, its mechanism remains unclear. Here, we further clarify the function of DUSP28 in pancreatic cancers. Analysis using a public microarray database and *in vitro* assay indicated a critical role of platelet derived growth factor A (PDGF-A) in pancreatic cancer malignancy. PDGF-A was positively regulated by DUSP28 expression at the mRNA and protein levels. Enhanced DUSP28 sensitized pancreatic cancer cells to exogenous PDGF-A treatment in migration, invasion, and proliferation. Transfection with siRNA targeting DUSP28 blunted the influence of administered PDGF-A by inhibition of phosphorylation of FAK, ERK1/2, and p38 signalling pathways. In addition, DUSP28 and PDGF-A formed an acquired autonomous autocrine-signaling pathway. Furthermore, targeting DUSP28 inhibited the tumor growth and migratory features through the blockade of PDGF-A expression and intracellular signaling *in vivo*. Our results establish novel insight into DUSP28 and PDGF-A related autonomous signaling pathway in pancreatic cancer.

## Introduction

Pancreatic cancer is one of the most lethal malignancies with a 5-year survival rate <7%^1,2^. The poor prognosis results from the difficulty of initial diagnosis, strong migratory nature, and high resistance to conventional cancer therapies^3–6^. It is the only cancer in which the prognosis has remained unchanged despite significant advances in medical therapy and surgical techniques during the past few decades^7,8^. Effective suppression of pancreatic cancers is urgently required.

Dual-specificity phosphatases (DUSPs) are protein phosphatases that tune the activities of mitogen-activated protein kinases (MAPKs). DUSPs are critical in regulating cancer cell growth and survival^9,10^. To date, 25 DUSP genes have been listed in Human Genome Organization databases. Based on de-phosphorylation or phosphorylation, these genes can be divided as “classical” or “non-classical” concerning specific residues in various cancer cells^11–14^. Although we have previously reported that blockade of DUSP28 suppresses chemo-resistance and migration in pancreatic cancer through the inhibition of the extracellular signal-regulated kinase 1/2 (ERK1/2) signaling pathway^15^, its underlying mechanism remains elusive.

Natural cues, such as autocrine/paracrine growth factor signaling, hypoxia, extracellular matrix, and interactions with neighboring cells orchestrate tumorigenesis and aggressiveness of pancreatic cancer cells in the tumor micro-environment. The constantly evolving tumor micro-environment is rich in growth factors and receptor tyrosine kinases (RTKs) that amplify a cascade of signaling pathways, leading to metastasis, proliferation, angiogenesis, resistance to cell-death, and the epithelial mesenchymal transition (EMT)^16–18^. Platelet-derived growth factor-A (PDGF-A) is one of the most functional growth factors, which is critical for pancreatic cancer progression and is correlated with poor prognosis^19^. The PDGF-A signaling pathway leads to proliferation, migration, angiogenesis, and metastasis^20,21^. Targeting the PDGF-A signaling pathway has been reported to induce anti-cancer effects in human pancreatic cancer^22,23^. Therefore, inhibition of PDGF-A signaling pathway has been suggested as a potential therapy for pancreatic cancer patients.

PDGF-A expression is regulated by mucin 1 (MUC1) during pancreatic cancer progression^24^. Expressions of c-MET and integrin-gamma 1 (ITG-α1) are fine-tuned by over-expressed DUSP12 *in vitro* ^24^. Interestingly, we have also reported that DUSP28 regulates the expressions of MUC5B and MUC16 to link malignancy in a pancreatic cancer cell line^25^. The collective observations indicate that various donor molecules are functionally correlated with their partners in pancreatic cancer cells.

In this study, we explored the unique role of DUSP28 in pancreatic cancer malignancy by inducing PDGF-A signals. Our novel results demonstrate that DUSP28 plays a pivotal role in pancreatic cancer malignancy, suggesting that targeting DUSP28 might be a smart strategy for blocking pancreatic cancer.

## Results

### Functional PDGF-A activates malignancy of human pancreatic cancer cells

**Figure 1:**
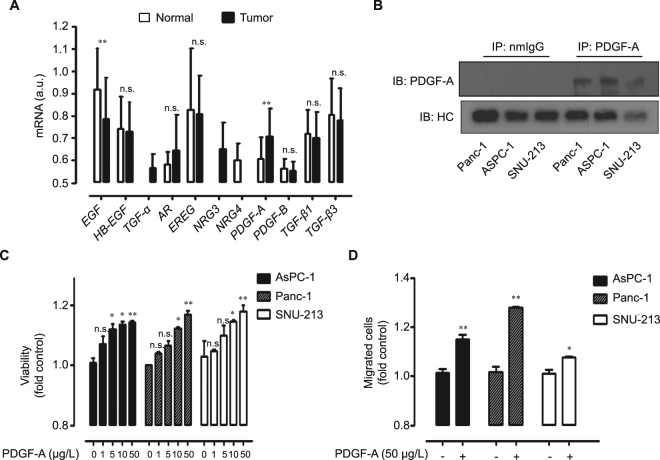
Functional platelet-derived growth factor (PDGF)-A is expressed in human pancreatic cancer. (**A**) Transcriptional levels of various growth factors in pancreatic cancer and normal pancreatic samples were analyzed using the Gene Expression Omnibus database (*P*-value by Student’s *t* test, **p < 0.01, a.u. indicates arbitrary units for the UPC method, n.s., non-significant) (**B**) Panc-1, AsPC-1, and SNU-213 cells were incubated for 72 h then cultured supernatants were used for immunoprecipitation using polyclonal platelet-derived growth factor (PDGF)-A and normal mouse IgG antibodies. Soluble PDGF-A was subjected to Western blot analysis using monoclonal antibody specific for PDGF-A. Heavy chain (HC) of antibodies was used as a control. (**C**) Three human pancreatic cancer cell lines were incubated with various doses of PDGF-A in serum free cultured medium for 72 h. Viability was measured by the WST-1 assay (*n* = 3; Tukey’s *post hoc* test was applied to detect differences in ANOVA, *p* < 0.0001; asterisks indicate a significant difference compared with 0% inhibition, *p < 0.05, **p < 0.01, n.s., non-significant) (**D**) Three human pancreatic cancer cell lines were incubated with 50 µg/L PDGF-A for 6 h. Migration was evaluated using the Transwell migration assay (*n* = 3; Tukey’s *post-hoc* test was applied to detect significant group difference in ANOVA, *p* < 0.0001; asterisks indicate a significant difference compared with 0% inhibition, *p < 0.05, **p < 0.01).

We previously demonstrated that DUSP28 has a functional role in drug resistance and migratory activity, and tunes the expression of pivotal molecules including MUC5B and MUC16 in human pancreatic cancers^15,25^. Presently, we further investigated the functional correlation between DUSP28 and specific growth factors as natural cues to aggravate the malignancy of pancreatic cancers. To determine the prominent growth factors in human pancreatic cancer, various growth factor mRNA expression profiles were examined using the Gene Expression Omnibus (GEO) public microarray database. Analysis of the mRNA profiles suggested that PDGF-A signaling might be important in pancreatic cancers (Fig. 1A). The role of PDGF-A was assessed in human pancreatic cancer cells. Expression levels of soluble PDGF-A in cell culture media were examined using immuno-precipitation. Soluble PDGF-A expressions were relatively different in Panc-1, AsPC-1, and SNU-213 cells, respectively (Fig. 1B). Human recombinant PDGF-A was added exogenously to cultures of AsPC-1, Panc-1, and SNU-213 cells. Exposure to PDGF-A produced similar effects, with dose-dependent increases of viability of the three cell types compared to control cells treated with phosphate-buffered saline (Fig. 1C). Treatment with 50 µg/L PDGF-A significantly induced cell migration in AsPC-1 and Panc-1 cells, but only marginally in SNU-213 cells (Fig. 1D).

### DUSP28 regulates PDGF-A expression

**Figure 2:**
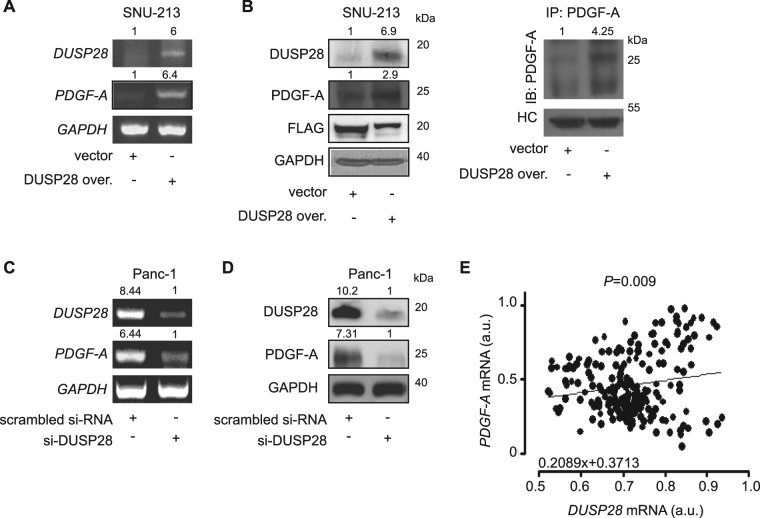
Dual-specificity phosphatase28 (DUSP28) positively regulates platelet-derived growth factor (PDGF)-A expression. (**A**) SNU-213 cells were transfected with a control plasmid or the pcDNA-DUSP28 construct. *PDGF-A* mRNA levels were analyzed by reverse-transcription polymerase chain reaction (RT-PCR). *Glycerladehyde-3-phosphate dehydrogenase* (*GAPDH*) was used as a control. (**B**) SNU-213 cells were transfected with a control plasmid or a pcDNA-DUSP28 construct. PDGF-A and soluble PDGF-A protein levels were analyzed by Western blot (left) and immunoprecipitation (right), respectively. GAPDH, FLAG, and heavy chain (HC) of antibodies was used as a control. (**C**) Panc-1 cells were transfected with scrambled or DUSP28-specific siRNA. After 72 h of transfection, *PDGF-A* mRNA levels were analyzed by reverse-transcription polymerase chain reaction (RT-PCR). *GAPDH* was used as a control. (**D**) Panc-1 cells were transfected with scrambled or DUSP28-specific siRNA. After 72 h of transfection, PDGF-A protein levels were analyzed by Western blot. GAPDH was used as a control. (**E**) The correlation between *PDGF-A* and *DUSP28* expressions in pancreatic cancer samples was calculated using the Gene Expression Omnibus (GEO) public microarray database (Pearson’s correlation coefficient (PCC) was used for statistical analysis).

To investigate the correlation between DUSP28 and PDGF-A expression in human pancreatic cancer, we examined PDGF-A expression following DUSP28 over-expression in SNU-213 cells, which poorly express DUSP28. Enhanced expression of DUSP28 increased PDGF-A expression at the mRNA and cellular protein levels in SNU-213 cells compared to control plasmid-transfected SNU-213 cells. In addition, soluble PDGF-A protein was also highly increased in culture medium of DUSP28-overexpressing SNU-213 cells compared to control cells (Fig. 2A,B). In contrast, mRNA and cellular protein levels of PDGF-A were down-regulated by si-DUSP28 transfection in Panc-1 cells that strongly expressed DUSP28 (Fig. 2C,D). To confirm the involvement of DUSP28 expression in PDGF-A induction, we analyzed the pancreatic cancer GEO public microarray database. A positive correlation was found between DUSP28 and PDGF-A expression in pancreatic cancer samples (Fig. 2E), with a negative correlation between PDGF-A and DUSP28 in normal pancreas samples (data not shown). These results suggest that PDGF-A expression may be regulated by DUSP28 expression.

### Effect of PDGF-A is dependent on DUSP28 expression in human pancreatic cancer cells

**Figure 3:**
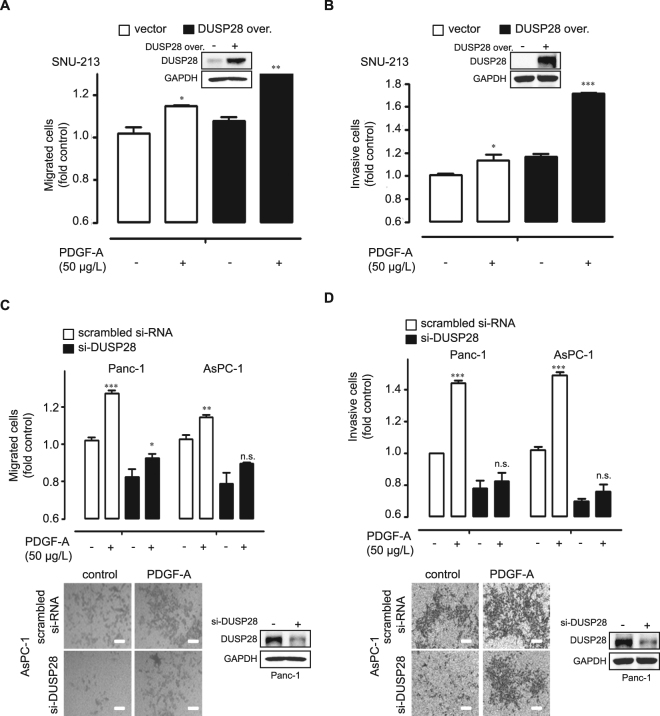
Effect of platelet-derived growth factor (PDGF)-A treatment is dependent on dual-specificity phosphatase28 (DUSP28) expression in human pancreatic cancer cells. (**A**,**B**) SNU-213 cells were transfected with a control plasmid or a pcDNA-DUSP28 construct. After 72 h of transfection, the cells were exposed to a serum-starved condition. After 18 h of serum-starvation, SNU-213 cells were additionally incubated with PDGF-A (50 μg/L) for 6 h to assess migration or for 24 h to invasion activity, respectively. Migrated or invasive cells were evaluated using the Transwell-migration or -invasion assay. DUSP28 levels were significantly increased in SNU-213 cells after DUSP28 overexpression (In let). (*n* = 3; Tukey’s *post-hoc* test was applied to detect significant differences in ANOVA, *p* < 0.0001; asterisks indicate a significant difference compared with 0% inhibition, *p < 0.05, **p < 0.01, ***p < 0.001). (**C**,**D**) Top, Panc-1 and AsPC-1 cells were transfected with scrambled or DUSP28-specific siRNA. Migration and invasion activities were assayed as described above. Bottom, representative image of a Trans-well migration or -invasion assay of AsPC-1 cells (scale bar = 50 µm). DUSP28 levels were significantly decreased in Panc-1 cells after si-DUSP28 transfections (In let).

To demonstrate the role of DUSP28 expression to exogenous PDGF-A treatment in human pancreatic cancer, we examined the viability, migration, and invasion activities of pancreatic cancer cells following regulation of DUSP28 expression. Overexpression of DUSP28 significantly promoted migratory and invasive activity of SNU-213 cells exogenously treated with PDGF-A compared to control plasmid transfected cells (Fig. 3A,B). PDGF-A also significantly induced proliferation of DUSP28 enhanced SNU-213 cells compared to control plasmid transfected SNU-213 cells, though the difference was weak (Supplementary Fig. 1A). In addition, blocking DUSP28 expression significantly diminished migratory and invasive activities in AsPC-1 and Panc-1 cells stimulated by exogenous PDGF-A compared to those in scrambled si-RNA transfected cells (Fig. 3C,D). Transfection of si-DUSP28 also blunted viability in AsPC-1 and Panc-1 cells stimulated by exogenous PDGF-A compared to scrambled si-RNA transfected cells (Supplementary Fig. 1B).

**Figure 4:**
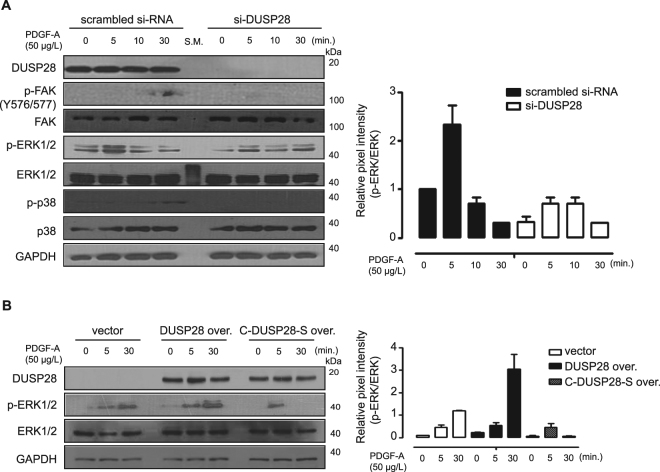
Intracellular signaling activated by platelet-derived growth factor (PDGF)-A is dependent on dual-specificity phosphatase28 (DUSP28) expression in human pancreatic cancer cells. (**A**) AsPC-1 cells were transfected with scrambled or DUSP28-specific siRNA. After 48 h of transfection, the cells were exposed to a serum-starved condition. After 18 h of serum-starvation, AsPC-1 cells were incubated with PDGF-A (50 μg/L) for various times, and the cell lysates were subjected to Western blot analysis using antibodies specific for DUSP28, phospho-FAK (Y576/577), total FAK, phospho-ERK1/2 (T202/204), total ERK1/2, phospho-p38 (S473), total p38, and GAPDH. Intensity of pERK/ERK1 was measured using ImageJ software. (**B**) SNU-213 cells were transfected with a control plasmid, a pcDNA-DUSP28 construct, or a pcDNA-C103S DUSP28 construct. After 48 h of transfection, the cells were exposed to a serum-starved condition. After 18 h of serum-starvation, SNU-213 cells were incubated with PDGF-A (50 μg/L) for different time periods, and the cell lysates were subjected to Western blot analysis using antibodies specific for DUSP28, phospho-ERK1/2 (T202/204), total ERK1/2, and GAPDH. Intensity of pERK/ERK1 was measured using ImageJ software.

To elucidate the involvement of DUSP28 expression in intracellular signaling activated by PDGF-A, we examined the signal transduction pathways stimulated by PDGF-A treatment in scrambled si-RNA-and si-DUSP28-transfected AsPC-1 cells. Treatment with PDGF-A significantly decreased tyrosine phosphorylation in a time-dependent manner in si-DUSP28-transfected AsPC-1 cells compared to scrambled si-RNA-transfected cells (Supplementary Fig. 2). To further analyze the detailed mechanism of DUSP28 expression in AsPC-1 cells, phosphorylation levels of various molecules were examined under the same conditions. Treatment with PDGF-A increased the phosphorylation level of FAK (Y576/577) in AsPC-1 cells, but not in si-DUSP28 transfected cells. PDGF-A also strongly induced ERK1/2 and p38 phosphorylation in scrambled si-RNA-transfected AsPC-1 cells, compared to si-DUSP28-transfected cells (Fig. 4A). Similar results were obtained in Panc-1 cells (data not shown). Next, we confirmed the regulation of intracellular signaling by DUSP28 expression using wild-type DUSP28 and mutant DUSP28-C103S overexpression in DUSP28-negative SNU-213 cells. The mutated DUSP28-C103S transfection had little activity compared to those of DUSP28-WT transfection^26^. Transfection with the wild-type DUSP28 construct induced a time-dependent increase in ERK1/2 phosphorylation compared to control plasmid or DUSP28-C103S construct-transfected cells (Fig. 4B). These results suggest that functional DUSP28 regulates the pancreatic cancer malignancy activated by PDGF-A treatment through the intracellular signaling.

### DUSP28 frames an autocrine loop with PDGF-A

**Figure 5:**
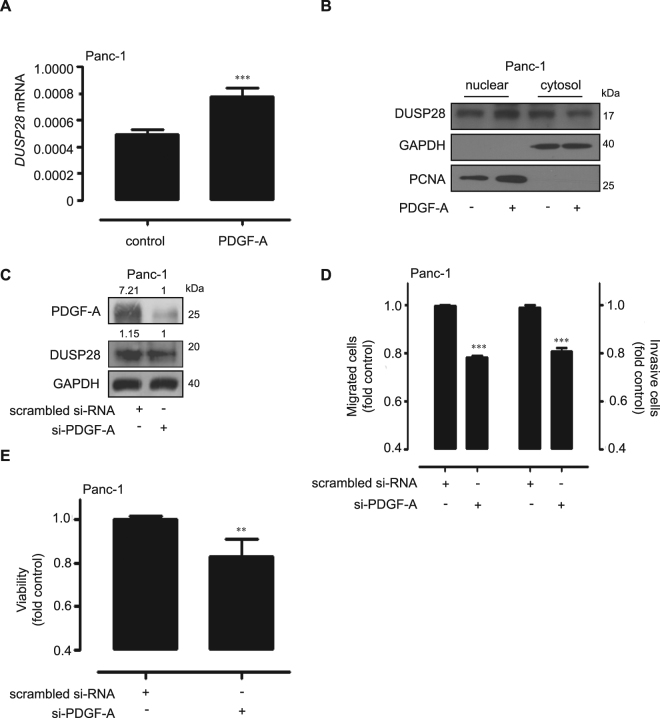
Functional dual-specificity phosphatase28 (DUSP28) and platelet-derived growth factor (PDGF)-A form an autocrine loop in human pancreatic cancer cells. (**A**) Panc-1 cells were incubated with 50 µg/L of PDGF-A for 24 h. *DUSP28* mRNA was evaluated by quantitative reverse-transcription polymerase chain reaction (qRT-PCR) (*n* = 3; Tukey’s *post-hoc* test was used to detect significant differences of ANOVA, *p* < 0.0001; asterisks indicate a significant difference compared with 0% inhibition, ***p < 0.001). (**B**) Panc-1 cells were incubated with 50 µg/L of PDGF-A for 24 h. Fractionated lysates (nuclear and cytosol fractions) were subjected to Western blot analysis for DUSP28. GAPDH and proliferating cell nuclear antigen (PCNA) were used to test the efficacy of the cytosolic and nuclear fractionation, respectively. (**C**) Panc-1 cells were transfected with scrambled or PDGF-A specific siRNA. After 72 h of transfection, PDGF-A and DUSP28 protein levels were analyzed by Western blot. GAPDH was used as a control. (**D**) Panc-1 cells were transfected with scrambled or PDGF-A specific siRNA. After 48 h of transfection, the cells were exposed to a serum-starved condition. After 18 h of serum-starvation, migrated or invasive cells were evaluated using the Transwell-migration or -invasion assay (*n* = 3; Tukey’s *post-hoc* test was applied to detect significant differences in ANOVA, *p* < 0.0001; asterisks indicate a significant difference compared with 0% inhibition, ***p < 0.001). (**E**) Panc-1 cells were transfected with scrambled or PDGF-A specific siRNA. Cell viability was measured by the WST-1 assay (*n* = 3; Tukey’s *post hoc* test was applied to detect differences in ANOVA, *p* < 0.0001; asterisks indicate a significant difference compared with 0% inhibition, **p < 0.01).

To demonstrate correlations between DUSP28 and PDGF-A in human pancreatic cancer, we examined the effect of PDGF-A treatment on DUSP28 expression in Panc-1 cells. Treatment with PDGF-A increased *DUSP28* mRNA expression levels in Panc-1 cells (Fig. 5A). We next compared the DUSP28 protein level in the cytosolic and nuclear fractions extracted from PDGF-A-treated Panc-1 cells. DUSP28 protein level of PDGF-A-treated Panc-1 cells was significantly higher in the nuclear fraction and lower in the cytosolic fraction, compared to untreated Panc-1 cells (Fig. 5B), indicating that PDGF-A treatment affects both DUSP28 expression and localization. To prove the correlation between PDGF-A and DUSP28, we analyzed DUSP28 protein level following si-PDGF-A transfection in Panc-1 cells. As shown Fig. 5C, protein level of DUSP28 was affected by reduction of PDGF-A levels. To verify the significance of PDGF-A in pancreatic cancer malignancy, we used different types of si-PDGF-A transfection in Panc-1 cells. Transfection of si-PDGF-A inhibited migration, invasion, and viability of Panc-1 cells compared to scrambled si-RNA transfected cells (Fig. 5C–E). Similar results were obtained using AsPC-1 cells (data not shown). These results suggest that DUSP28 and PDGF-A form a unique autocrine loop that specifically affects pancreatic cancer malignancy.

### Blocking DUSP28 deadens human pancreatic cancer malignancy

We constructed a stable-reducible sh-DUSP28 Panc-1 cell line to investigate the role of DUSP28 *in vivo*. We first checked the expression levels and characteristics of established cell lines *in vitro*. Sh-DUSP28 Panc-1 cells significantly reduced DUSP28 expression and accompanied by limited migration, invasion, and proliferation activities (Supplementary Fig. 3A–E).

**Figure 6:**
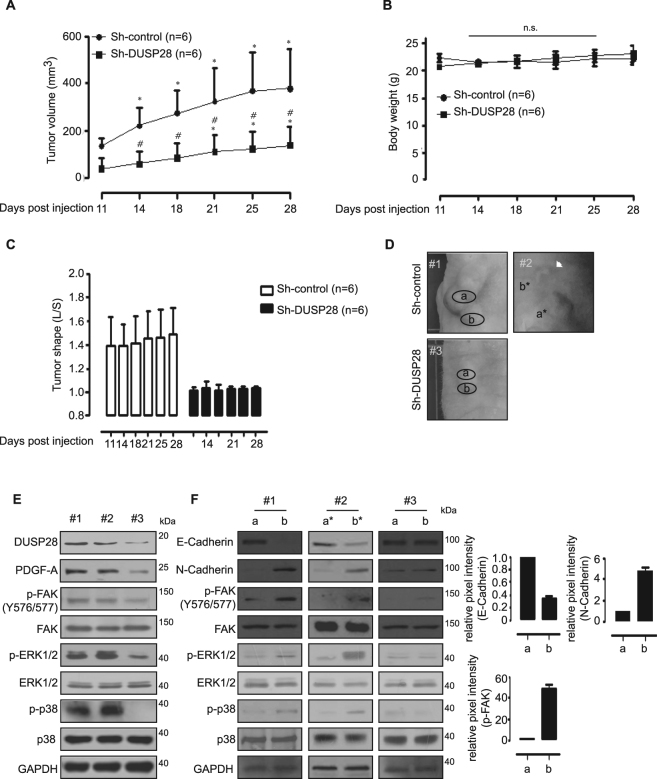
Targeting dual-specificity phosphatase28 (DUSP28) inhibits human pancreatic cancer malignancy *in vivo*. (**A**) Anti-tumor effects of sh-DUSP28 Panc-1 xenograft models (Sh-control group: *n* = 6, Sh-DUSP28 group: *n* = 6) were measured for 28 days using the formula: V = 0.523 LW^2^ (L = length, W = width) (Tukey’s *post-hoc* test was applied to detect significant differences in ANOVA, *p* < 0.0001; asterisks indicate a significant difference compared with 0% inhibition, *p < 0.05, ^**#**^ *p* < 0.05 compared between Sh-control group and Sh-DUSP28 group). (**B**) Body weight in each group was regularly measured. (**C**) Effects of DUSP28 expression on morphological features of the sh-control and sh-DUSP28 Panc-1 xenograft models (tumor shape is long/short axis of tumors). (**D**) Representative images of the sh-control (#1 and #2) and sh-DUSP28 (#3) Panc-1 xenograft models. White arrows indicate newly generated tumors from the tumor-headquarter. (**E**) The lysates from sh-control group (#1 and #2) and sh-DUSP28 group (#3) tumor samples were tested for DUSP28, PDGF-A, p-FAK, FAK, p-ERK1/2, ERK1/2, p-p38, p38 and GAPDH by Western blot analysis. (**F**) Lysates from the core (a) and peripheral regions (b) of the tumors (#1 and #3) and body (a*) and newly migrated (b*) of the tumor (#2) were used to assess for expressions of E-cadherin, N-cadherin, phospho-FAK (Y576/577), total FAK, p-ERK1/2, ERK1/2, p-p38, p38, and GAPDH by Western blot analysis. Intensities of E-Cadherin/GAPDH, N-Cadherin/GAPDH, and p-FAK/FAK in #1 tumor model were measured using ImageJ software.

The effect of reduced DUSP28 was observed in a sh-DUSP28 and sh-control Panc-1 xenograft models. Control tumors grew to a mean size of 379.02 ± 163.77 mm^3^ after 25 days transplantation, whereas the shRNA-reducible tumors remained a mean size of 157.30 ± 75.04 mm^3^ after 25 days transplantation (Fig. 6A). No weight loss was detected in the control and sh-DUSP28 groups of the Panc-1 xenograft models (Fig. 6B). Tumor shapes of sh-DUSP28 tumor models (long/short axis) were close to 1 compared to the control group expressing DUSP28 (Fig. 6C), indicating that DUSP28 expressed tumors seem to have longish characteristic compared to DUSP28 reduced tumors. In addition, we investigated the roles of DUSP28 in the pancreatic cancer migratory characteristics using morphological changes and Western blot analysis. We divided the representative xenograft models into three groups (sh-control; #1 and #2; sh-DUSP28; #3). #2 model of sh-control group showed newly migrated tumors (b*) in the distant area from the injection site (a*) at a rate of 33% (two of six cases). We designated the core and peripheral regions of the tumor models as a and b, respectively (Fig. 6D). Then, we performed the western blot analysis using whole fractions of #1, #2, and #3 samples. DUSP28, PDGF-A, phospho-FAK, phospho-ERK1/2, and phospho-p38 levels were significantly decreased in sh-DUSP28 Panc-1 xenograft models (#3) compared to those of sh-control (#1 and #2) (Fig. 6E). In addition, we performed the western blot analysis using each fraction of a and b in #1, #3 and a* and b* in #2 models using antibodies against E-cadherin and N-cadherin, which are representative molecules of the EMT, along with phospho-FAK. In two tumors expressing DUSP28 (#1 and #2), levels of the N-cadherin and phospho-FAK, phospho-ERK1/2, and phospho-p38 proteins were quite higher in b fractions than those in a fractions, whereas no difference was detected between a and b fractions in DUSP28-reduced tumor sample (#3) (Fig. 6F). These results indicate that the pancreatic cancer malignancy is affected by the DUSP28 expression levels *in vivo*.

## Discussion

The present findings demonstrate that PDGF-A signaling pathway has critical roles in pancreatic cancer, and that its expression is regulated by functional DUSP28 at the mRNA and protein levels. Furthermore, DUSP28 and PDGF-A form a unique autocrine loop that specifically affected the pancreatic cancer malignancy *in vitro* and *in vivo* through acquired intracellular signaling. This is the first report to our knowledge in that targeting DUSP28 might be a promising new therapeutic approach to inhibit the malignant pancreatic cancers.

For the current study, we adopted the *in silico* comparison system to investigate the actual molecules involved using analysis of the pancreatic cancer database compared with those in the normal pancreas^25,27^. Analysis using the GEO public microarray database showed pancreatic cancer cells have significantly high mRNA expression of *PDGF-A* compared to cells in normal healthy pancreas. As proof-of-concept, exogenous PDGF-A treatment activated the migration, invasion, and proliferation in pancreatic cancers *in vitro*.

Functional growth factors have been linked to an increase in pancreatic cancer malignancy^28,29^. Presently, the expression of *PDGF-A* was positively accompanied by DUSP28 expression using the computation of *PDGF-A* mRNA levels as *DUSP28* expressions in GEO public microarray database and *in vitro* RT-PCR and Western blot analysis. This is the first report of DUSP28-related upregulation of oncogenic PDGF-A in pancreatic cancer cells.

We also verified the effectiveness of the correlation of DUSP28 expression and PDGF-A treatment in pancreatic cancer. If DUSP28 only upregulates functionally soluble PDGF-A expression levels, the exogenous PDGF-A treatment should complement the anti-cancer effects of si-DUSP28 transfection. However, exogenous treatment with PDGF-A could not completely compensate for the anti-cancer effects of si-DUSP28 transfection. Therefore, the anti-cancer effects of si-DUSP28 expression seem to be not only dependent on the reduction of soluble form of PDGF-A, which are usually regarded as the leading mechanisms for PDGF-A action, but also on the decline of cellular PDGF-A expression apart from previous findings^30^.

Most notably, DUSP28 and PDGF-A were shown to form a unique autocrine loop that specifically affects the pancreatic cancer malignancy *in vitro* and *in vivo* through acquired intracellular signaling as self-sufficient armament modules. Given that DUSP28 is significantly overexpressed in pancreatic cancers^15^, our results uniquely suggest that this autocrine loop might be involved in autonomous pancreatic cancer malignancy.

Of interest, remarkable anti-cancer effects of targeting DUSP28 were observed *in vivo* models. We have previously reported the differential sensitivity to gemcitabine, currently used anti-pancreatic cancer drug as DUSP28 expression levels in human pancreatic cancer *in vivo* models^15^. In the present study, remarkable anti-cancer effects of targeting DUSP28 were firstly observed *in vivo* models through the convincing inhibition of intracellular signaling. PDGF-A is highly expressed in pancreatic cancer and is associated with poor prognosis^19,29,31^. In agreement with these reports, our results support that low expression of PDGF-A could improve the clinical outcomes in pancreatic cancer patients. DUSP28 also improves the clinical outcomes in patients with pancreatic cancer though the difference was not statistically significant.

In summary, our results unveil the mechanisms underlying autonomous malignancy induced by natural cues in pancreatic cancers and suggest that DUSP28, an executor of a unique autocrine loop, could be a target molecule to inhibit pancreatic cancer malignancy. It also provides further insight for treatment of malignant pancreatic cancer.

## Materials and Methods

### Gene expression analysis

Microarray expression profiles were obtained from the Gene Expression Omnibus (GEO) public microarray database. We integrated data sets independently obtained from several research groups using the absolute normalization method SCAN.UPC^25^. We restricted the integration to Affymetrix Human Genome U133 Plus 2.0 Array platform (GPL570) because the normalization method is dependent on the total number of probes and GPL570 has more probes than GPL96 and GPL97. All data were normalized by the default option of SCAN.UPC. The histological type of each sample was assigned according to sample annotation in GEO. In total, eight data sets were used: GSE9599, −15471, −16515, −17891, −32676, −39409, −42952, and −46385.

### Cell culture and reagents

AsPC-1, Panc-1, and SNU-213 human pancreatic cancer cell lines were purchased from the Korean Cell Line Bank (Seoul, Korea). The cells were grown as described previously^27^. Antibodies for DUSP28 and PDGF-A were from Santa Cruz Biotechnology (Santa Cruz, CA, USA) and those for phospho-focal adhesion kinase (FAK), phosphor-FAK (Tyr576/577), phospho-ERK1/2 (Thr202/204), ERK1/2, phospho-p38 (Ser473), p38, and glyceraldehyde-3-phosphate dehydrogenase (GAPDH) were purchased from Cell Signaling Technology (Beverly, MA, USA). Recombinant soluble PDGF-A was obtained from R&D Systems (Minneapolis, MN, USA).

### Small interfering (si)RNA transfection

siRNA transfection was performed using an effectene (Qiagen, Hilden, Germany) as described previously^32^. Oligonucleotides specific for *DUSP28* (sc-94445 and 1120164a/b) and *PDGF-A* (sc-39703 and 1114379) were obtained from Santa Cruz Biotechnology and Bioneer (Daejeon, Korea), respectively. The scrambled control (sc-37007) was purchased from Santa Cruz Biotechnology.

### Measurement of cell viability

To evaluate cell viability with PDGF-A treatment, WST-1 reagent (Nalgene, Rochester, NY, USA) was used as described previously^33^. After a 10-min incubation at room temperature, the absorbance was measured at 450 nm using a microplate reader (Bio-Rad, Richmond, CA, USA).

### Migration assay

Migration assays were performed using 24-Transwell plates (Corning, Corning, NY, USA) according to the supplier’s protocol^34^. Cells were applied to the upper-chamber containing RPMI without serum for 6 h. Cells that had migrated to the back side of the filter after 24 h were stained. The eluted dye was measured at 560 nm in an enzyme-linked immunosorbent assay (ELISA) reader (Bio-Rad).

### Invasion assay

Invasion assays were performed using growth factor-reduced Matrigel (BD Biosciences, San Diego, CA, USA) coated on 24-Transwell plates according to the supplier’s protocol. Cells were applied to the upper-chamber containing RPMI without serum for 24 h. Cells that had migrated to the back side of the filter after 24 h were stained. The eluted dye was measured at 560 nm in an ELISA reader.

### RNA preparation, reverse transcript, and quantitative real-time polymerase chain reaction

Total RNA was extracted using Trizol reagent (Invitrogen, Carlsbad, CA, USA) and converted to cDNA using a Takara RNA PCR kit (Takara Bio Inc, Shiga, Japan) as described previously^25^. RT-PCR and quantitative real-time PCR analysis were performed using a Takara RT-PCR kit and StepOne Real-Time PCR system (Applied Biosystems) according to the manufacturers’ protocols. Primers for human *DUSP28* were obtained from Bioneer, and the *PDGF-A* and *GAPDH* gene primers were designed using Primer Express 3.0 (Applied Biosystems). The primer sequences were *DUSP-28*, forward: 5′-GCGGGATCCATGGGACCGGCAGAAGCTGGGCG-3′; reverse: 5′-CCCGCTCGAGTCAAGCCTCAGGGCCCAACCCTAA-3′ *PDGF-A*, forward 5′-GCAAGACCAGGACGGTCATTT-3′; reverse 5′-GGCACTTGACACTGCTCGT-3′ *GAPDH*, forward: 5′-TCACTGGCATGGCCTTCCGTG-3′; reverse: 5′-GCCATGAGGTCCACCACCCTG-3′.

### DUSP28 overexpression plasmid construct

Plasmids expressing FLAG-tagged *DUSP28* were constructed to overexpress DUSP28 in human pancreatic cancer cells, as described previously^15^.

### Western blot

Western blotting was performed to evaluate phosphorylation levels, as described previously^35^. Briefly, si-DUSP28-transfected cells were stimulated with PDGF-A for the indicated times. Bands were measured by densitometry using ImageJ software (National Institutes of Health, Bethesda, MD, USA).

### Immunoprecipitation assay

SNU-213 cells were transfected with control pcDNA3.1 plasmid and wild-type DUSP28 overexpressing construct in 60-mm dishes. After 4 days, the cultured supernatants were collected. Aliquots of the supernatants were incubated with anti-PDGF-A antibodies or normal IgG antibodies, and Protein G agarose was added (Amersham Biosciences, Little Chalfont, UK). Bound proteins were analyzed using Western blotting.

### Cell fractionation

The Nuclear/Cytosol Fractionation Kit (Bio Vision, Mountain View, CA, USA) was used according to the manufacturer’s instruction as described previously^32^. The cytoplasmic fractions were probed with GAPDH and the nuclear fractions were probed with proliferating cell nuclear antigen.

### Generation of the DUSP28-specific short hairpin (sh)RNA stable cell line

Plasmids expressing shRNAs and control shRNAs were obtained from Santa Cruz Biotechnology to stably suppress DUSP28 expression using a sh-activated gene silencing vector system. One day after transfection with shRNA constructs, Panc-1 cells were grown in Dulbecco’s complete medium containing 5 µM puromycin for 3 days to select the stable transfectants.

### Xenograft tumor model

Balb/c nude mice 6–8 weeks of age were obtained from Orient Bio (Seongnam, Korea). sh-control/Panc-1 (1 × 10^7^) and sh-DUSP28/Panc-1 cells (1 × 10^7^) were injected subcutaneously into the mouse flank as described previously^15^. Tumor volume was calculated as 0.523 LW^2^ (L = length, W = width). Tumor shape and body weight were recorded regularly. Animal care and experiments were carried out in accordance with guidelines approved by the animal bioethics committee of Jeju National University (2016-0049).

### Statistical analyses

Data are presented as mean ± standard deviation. Student’s *t-*test was used to compare two independent samples. Groups were compared by one-way analysis of variance followed by Tukey’s *post-hoc* test to identify significant differences using SPSS 12.0 K for Windows software (SPSS Inc., Chicago, IL, USA). A *p*-value < 0.05 was considered significant.

## Electronic supplementary material

Dataset 1
