# Supplementary material for: Autocrine DUSP28 signaling mediates pancreatic cancer malignancy via regulation of PDGF-A
Source: Sci Rep. 2017 Oct 6;7:12760. doi: 10.1038/s41598-017-13023-w (PMC5630619; doi:10.1038/s41598-017-13023-w)
Supplement: Supplementary file 1 — Dataset 1 [file 41598_2017_13023_MOESM1_ESM.doc]

**Autocrine DUSP28 signaling mediates pancreatic cancer malignancy via regulation of PDGF-A**

**Authors and Affiliations**

Jungwhoi Lee^1^*, Jungsul Lee^2^, Jeong Hun Yun^1^, Chulhee Choi^2^, Sayeon Cho^3^, Seung Jun Kim^4^, and Jae Hoon Kim^1^*

**Supplementary Fig. 1.**

**Effect of platelet-derived growth factor (PDGF)-A treatment is dependent on dual-specificity phosphatase28 (DUSP28) expression in human pancreatic cancer cells**

(A) Left, SNU-213 cells were transfected with a control plasmid or a pcDNA-DUSP28 construct. After 72 h of transfection, DUSP28 and GAPDH protein levels were analyzed by Western blot. Right, SNU-213 cells were transfected with a control plasmid or a pcDNA-DUSP28 construct. After 48 h of transfection, the cells were were incubated with PDGF-A (50 μg/L) in serum starved condition for additional 48 h. Cell viability was measured by the WST-1 assay (*n* = 3; Tukey’s *post hoc* test was applied to detect differences in ANOVA, *p* < 0.0001; asterisks indicate a significant difference compared with 0% inhibition, n.s., non-significant). (B) Left, Panc-1 and AsPC-1 cells were transfected with scrambled or DUSP28-specific siRNA. After 72 h of transfection, DUSP28 and GAPDH protein levels were analyzed by Western blot. Right, Cell viability was assessed described as above.

**Supplementary Fig. 2.**

**Intracellular signaling activated by platelet-derived growth factor (PDGF)-A is dependent on dual-specificity phosphatase28 (DUSP28) expression in human pancreatic cancer cells**

AsPC-1 cells were transfected with scrambled or DUSP28-specific siRNA. After 48 h of transfection, the cells were exposed to a serum-starved condition. After 18 h of serum-starvation, AsPC-1 cells were incubated with PDGF-A (50 μg/L) for various time periods, and the cell lysates were subjected to Western blot analysis using antibodies specific for DUSP28 (Top) and phospho-tyrosine (Y99) (Bottom).

**Supplementary Fig. 3.**

***In vitro* characterization of the short hairpin-dual-specificity phosphatase28 (sh-DUSP28) Panc-1 cell line** (A-B) DUSP28 mRNA and protein levels were evaluated by quantitative reverse transcription-polymerase chain reaction (qRT-PCR) and Western blot analysis of sh-control and sh-DUSP28 Panc-1 cells (*P*-value by Student’s *t* test and are representative of three individual experiments, ****p* < 0.001). (C-D) Migrated or invasive cells were respectively evaluated using the Transwell assay for 6 h or 24 h in sh-control and sh-DUSP28 Panc-1 cells (*P*-value by Student’s *t* test and are representative of three individual experiments, **p* < 0.05, ****p* < 0.001). (E) Cell viability was measured by the WST-1 assay in sh-control and sh-DUSP28 Panc-1 cells under a serum-starved condition (*p*-value by Student’s *t* test and are representative of three individual experiments, **p* < 0.05).
